# Uncovering the gray zone: mapping the global landscape of direct-to-consumer businesses offering interventions based on secretomes, extracellular vesicles, and exosomes

**DOI:** 10.1186/s13287-023-03335-2

**Published:** 2023-05-04

**Authors:** Atiyeh Asadpour, Badrul Hisham Yahaya, Katrina Bicknell, Graeme S. Cottrell, Darius Widera

**Affiliations:** 1 Stem Cell Biology and Regenerative Medicine Group, School of Pharmacy, University of Reading, PO Box 226, Whiteknights, Reading, RG6 6AP UK; 2 Lung Stem Cell and Gene Therapy Group, Department of Biomedical Sciences, Advanced Medical and Dental Institute (IPPT), Universiti Sains Malaysia, Sains@Bertam, 13200, Kepala Batas, Penang, Malaysia; 3 School of Pharmacy, University of Reading, PO Box 226, Whiteknights, Reading, RG6 6AP UK; 4 Cellular and Molecular Neuroscience, School of Pharmacy, University of Reading, PO Box 226, Whiteknights, Reading, RG6 6AP UK

**Keywords:** Stem cells, Exosomes, Secretome, Extracellular vesicles, Direct-to-consumer-businesses, Community health, Regenerative medicine

## Abstract

**Background:**

The last decade has seen a significant increase in media attention, industrial growth, and patient interest in stem cell-based interventions. This led to a rise in direct-to-consumer businesses offering stem cell “therapies” for multiple indications with little evidence of safety and efficacy. In parallel, the use of stem cell secretomes as a substitute for stem cell transplantation has become an increasing trend in regenerative medicine with multiple clinical trials currently assessing their efficacy and safety profile. As a result, multiple businesses and private clinics have now started to exploit this situation and are offering secretome-based interventions despite the lack of supporting data. This poses significant risks for the patients and could lead to a credibility crisis in the field.

**Methods:**

Internet searches were used to locate clinics marketing and selling interventions based on stem cell secretomes, exosomes, or extracellular vesicles. Data were extracted from websites with a particular focus on the global distribution of the businesses, the cellular source of the secretome, the indication spectrum, and the pricing of the provided services. Lastly, the types of evidence used on the websites of the businesses to market their services were extracted.

**Results:**

Overall, 114 companies market secretome-based therapies in 28 countries. The vast majority of the interventions are based on allogenic stem cells from undisclosed cellular sources and skin care is the most marketed indication. The price range is USD99–20,000 depending on the indication.

**Conclusions:**

The direct-to-consumer industry for secretome-based therapies appears to be primed for growth in the absence of appropriate regulatory frameworks and guidelines. We conclude that such business activity requires tight regulations and monitoring by the respective national regulatory bodies to prevent patients from being conned and more importantly from being put at risk.

**Supplementary Information:**

The online version contains supplementary material available at 10.1186/s13287-023-03335-2.

## Background

Stem cells are capable of self-renewal and can differentiate into multiple lineages. In contrast to pluripotent stem cells (e.g., embryonic stem cells or induced pluripotent stem cells), adult stem cells are multipotent and thus cannot cross the germ layer boundary. Mesenchymal stem cells or mesenchymal stromal cells (MSCs) can be readily obtained from both human and animal sources and give rise to osteogenic, adipogenic, and chondrogenic cells [1]. After their initial discovery in 1968 [2], multiple claims have been made that MSCs might be able to cross the germ layer boundaries and differentiate into cell types such as neurons or pancreatic islet-like cells [3, 4]. However, functional differentiation into non-mesodermal cell types has never been demonstrated. Nevertheless, more than 1471 clinical trials have been registered on the ClinicalTrials.gov database as of November 2022 (data retrieved on 09/11/2022) with indications ranging from musculoskeletal disorders to cancer [5].

Surprisingly, despite their lack of ability to differentiate into non-mesodermal cells, the results of the clinical trials revealed a good safety profile and often efficacy even in disorders and conditions affecting the ectoderm and endoderm. Unfortunately, due to these encouraging results and a lack of regulatory framework, this enabled a global rise of direct-to-consumer businesses selling unproven and unlicenced MSC-based interventions [6–9]**.** In 2016, Turner and Knoepfler found 351 businesses engaged in direct-to-consumer marketing of stem cell interventions in the USA alone [6]. A 2021 follow-up study revealed 1480 US businesses operating 2754 clinics [9]. Importantly, there are multiple reports on severe adverse effects of such interventions including but not limited to septicaemia or complete blindness [10, 11]**.**

Nowadays, there is a general consensus that the mechanism underlying the regenerative capacity of MSCs is paracrine [12–15]. The paracrine effect of MSCs is largely attributed to a reduction of inflammation, immunomodulation, and subsequent activation of endogenous regeneration [16–20].

All these `bystander effects` are mediated by soluble paracrine factors and cargo of extracellular vesicles (EVs) (reviewed in [21] and [22]). EVs are released by all cell types and nowadays it is widely accepted that they represent a universal means of cell–cell communication [23]. Consequently, EVs are involved in a variety of biological processes, such as immune system regulation, tissue homeostasis, regulation of inflammatory processes, and normal ageing [24]. Microvesicles, exosomes, and apoptotic bodies are the three primary subtypes of EVs, and they differ from one another in terms of their biogenesis, release mechanisms, size, composition, and function [24]. Exosomes are small EVs (~ 70–150 nm) of endosomal origin, while microvesicles (100–1000 nm) originate from the plasma membrane [24, 25]. It is important to note that it is difficult to distinguish between microvesicles and exosomes at the experimental level. Therefore, the International Society of Extracellular Vesicles (ISEV) is suggesting in their 2018 guidelines `Minimal Information for Studies of Extracellular Vesicles (MISEV)` the use of the term "EV" instead of "exosomes" and "microvesicles" [26]. The cargo of EVs includes lipids, nucleic acids such as miRNAs and mRNAs, and proteins [27].

Multiple pre-clinical studies and a clinical treatment attempt of a Graft-versus-host disease conducted in 2014 have suggested that MSC-derived EVs have a regenerative potential similar to MSCs themselves [28–31]. Since then, the field has rapidly evolved and EVs are currently being used in a large number of clinical trials for a variety of indications. As of November 2022, a total of 433 secretome-based clinical trials had been registered on the ClinicalTrials.gov database (secretome: 18, exosomes: 288, EVs: 127; data retrieved on 09/11/2022).

Similar to the raise of direct-to-consumer businesses offering unlicensed and unproven stem cell-based interventions, companies now exploit the field of EVs and are already selling secretome, and EV-based interventions directly to consumers.

The aim of this study was to map the global landscape of direct-to-consumer businesses offering secretome, exosome, and EV-based interventions. We further assessed the range of indications, the current pricing of these treatments, and the types of evidence that are used to market the services.

## Methods

We gathered publicly available material from the websites of direct-to-consumer businesses and private clinics offering interventions based on stem cell secretomes.

We conducted over 50 Google search engine keyword searches, using terms including `exosome therapy`, `secretome therapy`, and `cell-free therapy`. We also searched for the geographic location of the businesses, stem cell types (e.g., `adipose`, `bone-marrow`, `autologous` and `allogenic`), the indication of the intervention (e.g., `autism`), and the pricing. Lastly, based on a similar approach published earlier for stem cell-based interventions [32], we extracted information on the forms of evidence (e.g. `clinical trials`, `patient testimonials`, `peer-reviewed publications`, or `patents`) for the safety and efficacy of the procedures. All searches were conducted between July and November 2022, making our study a snapshot of direct-to-consumer businesses and private clinics active at that time. Third-party companies supplying secretomes, exosomes, and EVs to clinics and businesses were excluded from the results.

## Results

**Fig. 1:**
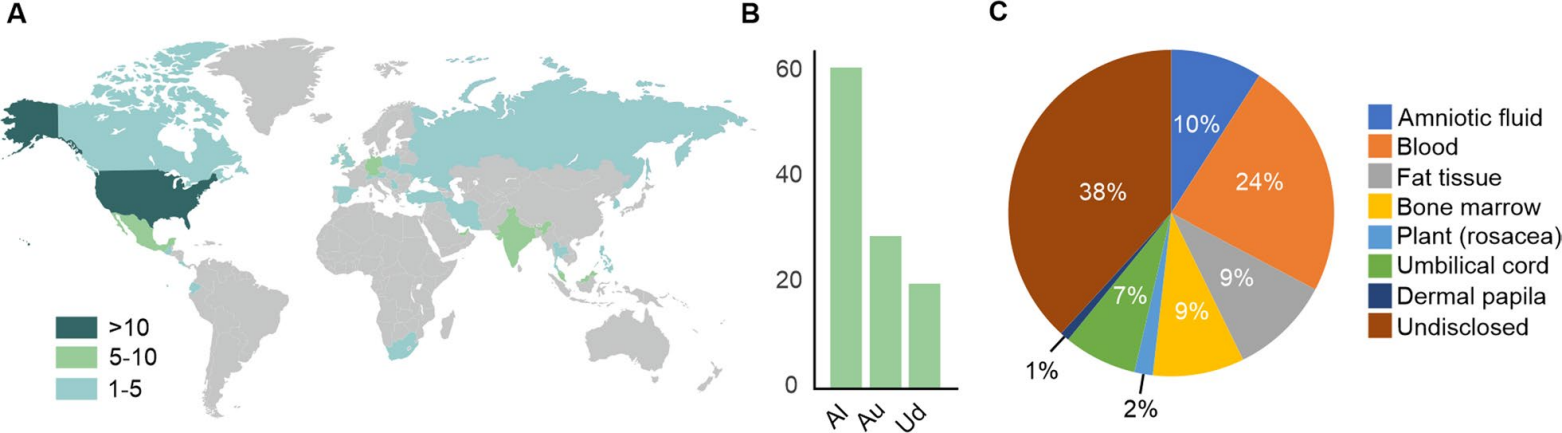
Global distribution of clinics and businesses providing secretome-based interventions and the source of the secretomes. Data were collected between July and November 2022 using Google as the search engine. **A** Distribution of direct-to-consumer businesses. **B** Type of stem cells used for the secretome generation. Al: allogenic; Au: autologous; Ud: undisclosed. **C** Source of stem cells used to generate the secretomes

We identified 114 businesses and private clinics marketing stem cell secretome-based interventions directly to consumers (Fig. 1A). Majority of the businesses and clinics were based in the USA (34) followed by Mexico (10), United Arab Emirates (8), and Malaysia (7). 5 businesses were operating in Germany and India, while 4 were found in Spain, the United Kingdom and Turkey. 3 businesses per country were identified in Canada, Thailand, Singapore, and Iran, whereas 2 were found in Serbia, the Russian Federation, Poland, and South Korea. Countries with 1 business or private clinic were Ireland, Switzerland, Slovenia, Austria, Ukraine, Guatemala, Costa Rica, Philippines, South Africa, Ecuador, and Cyprus. Some of the identified businesses offer secretome-based interventions on multiple sites across different countries.

63 businesses offered interventions involving secretomes from allogenic stem cells, 30 marketed secretomes from autologous cells, and 21 companies did not disclose this information (Fig. 1B). The tissue of origin of the cells was found to vary (Fig. 1C) with a majority of the businesses not disclosing the source (38%), followed by blood-derived cells (24%), amniotic fluid for 10%, adipose tissue (9%), the bone marrow (9%), and the umbilical cord (7%). Secretome from plant `stem cells` accounted for 2% and the dermal papilla for 1%, respectively.

**Fig. 2:**
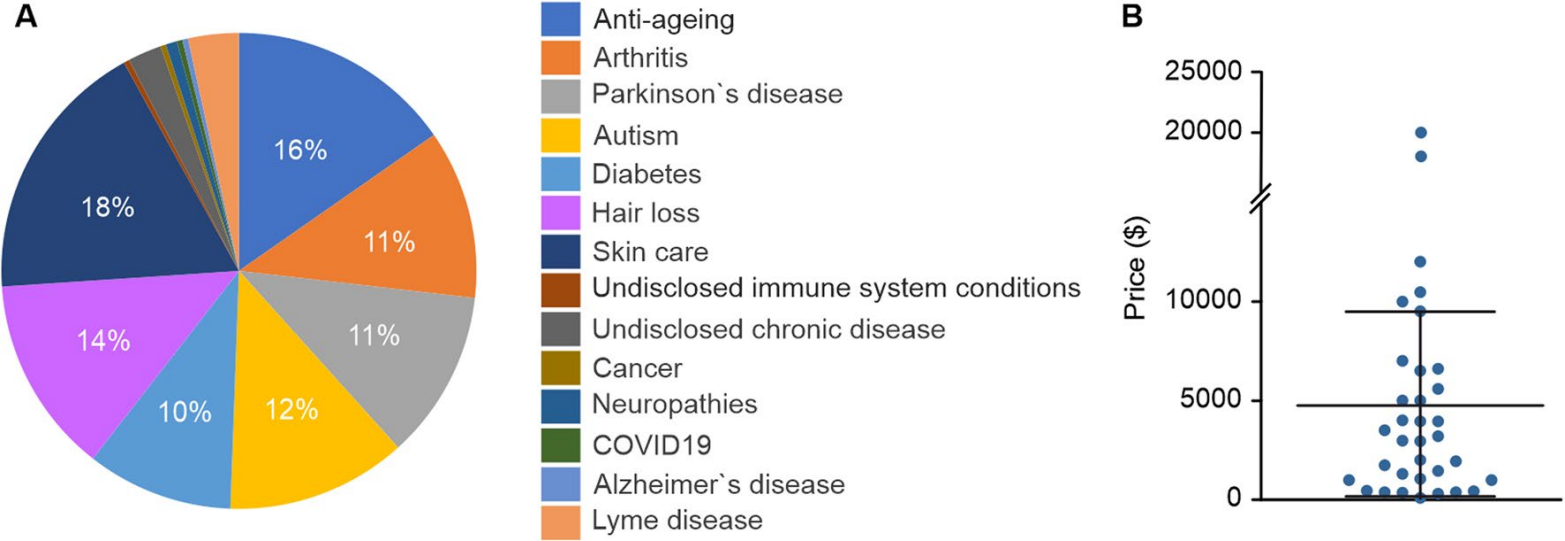
Indications and pricing for stem cell secretome-based interventions offered by the direct-to-consumer businesses. **A** Types of cosmetic or medical conditions treated by the direct-to-consumer businesses. **B** Pricing of the marketed interventions

The most frequently identified indication for the interventions was skin care (48 businesses) followed by anti-ageing (42), and hair loss (36). Interventions targeting autism were marketed by 32 businesses, whilst 30 have been offered for both arthritis and Parkinson`s disease. Other indications include diabetes (26), Lyme disease (9), undisclosed chronic diseases (6), neuropathies (2), undisclosed rare immune system conditions (1), COVID-19 (1), and Alzheimer`s disease (1) (Fig. 2A).

Most of the businesses and private clinics did not disclose the pricing on their websites and only indicated a price range without referencing the targeted condition. Overall, only 38 out of the 114 businesses and private clinics identified in this study include a price range on their websites. Analysis of the pricing revealed a wide spread of costs ranging from USD99-20,000 (Fig. 2B).

The lowest and highest prices for treatment are for skin care and arthritis, respectively and both were offered in the USA. In general, the pricing for cosmetic indications is at the lower end of the spectrum (~ USD100), while prices for severe conditions such as Parkinson`s disease, autism, or diabetes were in the higher region ranging from USD1310–20,000. To the best of our knowledge, all these interventions are not covered by the healthcare insurances and are thus out-of-pocket expenses.

**Fig. 3:**
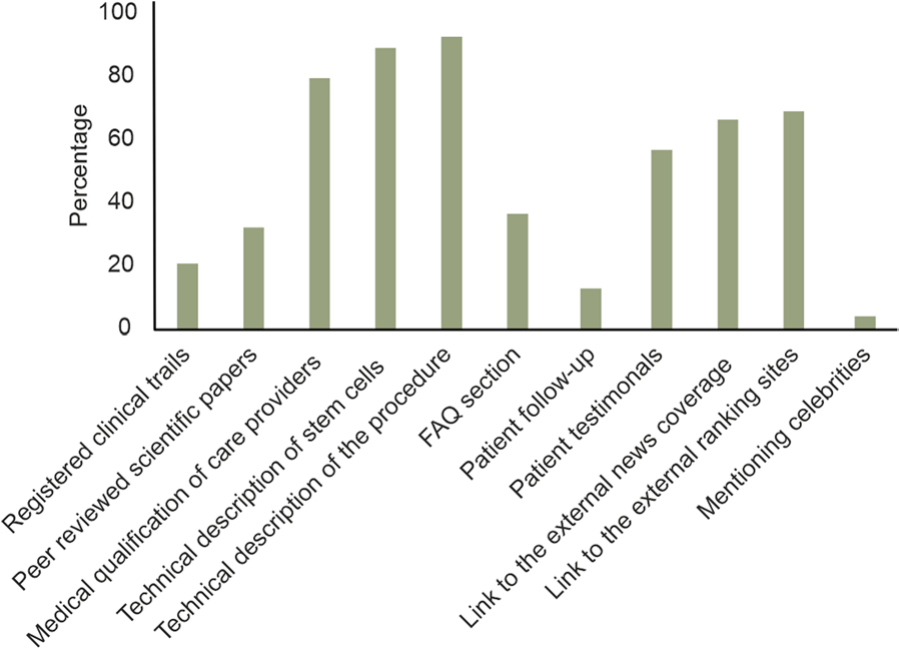
Forms of evidence used on the websites of the direct-to-consumer businesses marketing secretome therapies

Most businesses offering secretome therapies utilised weaker forms of evidence to market their interventions including technical description of the procedure, generic textbook-level information on stem cells and stem cell-secretome as well as medical qualifications of the care providers (Fig. 3). Most of the businesses (> 70%) used the medical qualifications of the healthcare providers, technical descriptions of the source of the secretomes/EVs and, technical descriptions of the procedures as the evidence, while only ~20 and 30% referred to registered clinical trials and peer-reviewed scientific papers respectively. Only a small percentage of the websites included references to celebrities (< 5%).

## Discussion

Despite considerable progress in the field, there are currently no regulatory-approved secretome, exosome, or EV-based therapies worldwide. The reasons for this are multifactorial and include:

I. *Lack of standardised manufacturing methods*: Secretomes and EVs can be isolated from various cell types, and their composition may vary depending on the source. Moreover, there are no standardised protocols for their production [33], which can lead to batch-to-batch variability and thus to inconsistency in their therapeutic effects.
II. *Limited understanding of the mechanisms of action*: Although secretomes and EVs have been shown to have therapeutic potential in various disease models [34] and anti-inflammatory and immunomodulatory effects, their exact mechanism of action is not fully understood [35]. This makes it challenging to develop appropriate potency assays and to optimise the therapeutic efficacy and safety [36].
III. *Regulatory challenges*: Developing secretome and EV-based therapies for human use requires navigating complex regulatory framework. As of February 2023, regulatory aspects of the use of secretome and EVs are not specified [37]. The lack of standardised production methods and limited understanding of their mechanisms of action can make it challenging to meet regulatory requirements, especially at the international level.
IV. *Scalability issues*: Secretome and EV production is often time-consuming, and it can be challenging to produce large quantities of the end product for clinical use. Moreover, the cost of manufacturing at scale can be prohibitively expensive.

For these reasons and due to the lack of data on the efficacy and safety profiles, all direct-to-consumer businesses marketing secretome-based interventions are offering unproven therapies. The marketing of secretome-based interventions is conducted mainly online and the clinics often operate in geographical locations without strict medical regulations. Surprisingly, our study revealed that the vast majority (> 30%) of the direct-to-consumer businesses offering secretome treatments are based in the USA where any novel treatment should be approved and regulated by the Food and Drug Administration (FDA). This is despite the public safety notification on exosome products released on the 6th December 2019 by the FDA [38]. In general, secretomes, EVs and exosomes used to treat diseases and conditions are regulated as drugs and biological products under the Public Health Service Act and the Federal Food Drug and Cosmetic Act and are subject to premarket review and approval requirements. The lack of action against the vast number of businesses offering EV-based therapies, despite the FDA`s 2019 warning [38] and the fact that no secretome and EV-therapies have FDA approval, could be explained by the large number of US businesses marketing stem cell-based therapies [9]. Enforcement actions against these businesses might be a higher priority given the potential for these therapies to harm consumers.

In total, 14 businesses are operating from the European Union (EU; regulated by the European Medicines Agency (EMA)), and 5 are based in the UK, where the regulatory body is Medicines and Healthcare products Regulatory Agency (MHRA). In the EU, similar interventions are defined as Advanced Therapy Medicinal Products (ATMPs) [39]. This involves a risk management plan and safety considerations whilst an assessment of the efficacy profile can be conducted in the post-authorisation phase. Thus, the substantial number of companies operating from the EU and the UK is surprising.

Interestingly, our research also showed that the number of businesses marketing secretome interventions is dynamic with several company websites removing references to stem cell secretomes whilst others have introduced it (data not shown). Therefore, our study is only a snapshot of the landscape of direct-to-consumer business offering this type of intervention between July and November 2022 (Additional file 1: Table S1).

Currently, there are no standard methods for isolating secretomes, EVs, and exosomes (reviewed in [33]). Consequently, the yield and purity of EVs and exosomes within the secretome vary depending on the method of isolation [40]. However, most businesses do not provide information about the isolation of the secretomes or purification of the EVs. Moreover, if not conducted under appropriate conditions, secretomes, and EV fractions can be contaminated by animal-derived components [41], antibodies used for the isolation [40], as well as microbial contaminants. Thus, unregulated application of stem cell secretomes poses significant health risks for the patients beyond the potential lack of efficacy. Notably, this has been recognised by the regulatory bodies and the scientific and medical community and as result both the FDA and the ISEV released patient information and safety notices warning to the general public on their website [38, 42].

Analysis of the route of application revealed that most businesses inject the secretomes or extracellular vesicles intravenously. However, the specificity of release and uptake of EVs is still not fully understood [43].

Our analysis revealed that, as expected, the costs of the secretome interventions offered by the businesses (up to £20,000) are out-of-pocket expenses. Thus, selling of such interventions generates additional economic burden, especially for patients suffering from currently uncurable conditions such as Parkinson`s or Alzheimer’s disease.

Our data also reveals that the evidence provided by the businesses for efficacy and safety of the interventions represents a mix of weak evidence such as patient testimonials which can be cherry-picked and some valid evidence where the interpretation is difficult for lay audience. This is in general agreement with the findings published recently by Cook and colleagues where a similar level of evidence has been reported for direct-to-consumer businesses offering stem cell interventions [32].

In their 2022 study, Guleria and colleagues assessed the current state of unproven cellular therapies across the globe [11]. In the same report, the authors eloquently suggested a workflow that can be used by patients and practitioners to evaluate proven and unproven cellular therapies. Based on these recommendations, we suggest the following steps to allow an informed decision on the services offered by businesses and clinics involved in marketing of direct-to-consumer interventions based on secretomes and EVs:

I. Consulting most up-to-date patient information and safety notices provided by the ISEV (https://www.isev.org/patient-information-and-safety-notice--extracellular-vesicles-exosomes-and-unproven-therapies).
II. Consulting of warnings provided by the regulatory bodies (e.g., FDA: https://www.cdc.gov/hai/outbreaks/stem-cell-products.html).
III. Critical evaluation of the forms of evidence used for marketing by the businesses (systematic research review with meta-analysis > registered complete clinical trials with results > ongoing registered clinical trial > peer reviewed original research reports).

Moreover, we urge the scientific community to engage in balanced communication with the lay community and educate the public not only about the promises of cutting-edge secretome and EV-based therapies, but also the dangers of unregulated and unproved therapies.

## Conclusions

Due to promising pre-clinical data and the successful use of EVs in first-in-man clinical applications, a substantial marketplace expansion is expected. This will unavoidably lead to an increase in globally operating businesses offering direct-to-consumer interventions for multiple indications, despite the lack of data on safety and efficacy. To protect the public from health and economic risks and avoid damage to the credibility of the scientific field, new policies are needed that will translate into a tight regulatory framework that is enforced locally.

We conclude that in addition to a tight regulatory framework, that is locally enforced, better communication with the public is needed.

Educating the general public about the risks associated with unproven therapies is crucial in raising awareness and reducing misinformation, ultimately helping to minimise harm to patients. This should include public debates, more patient information from the regulatory bodies, and campaigns warning the public about the risks of unproven secretome-based therapies.

## Supplementary Information

**Additional file 1**. **Supplementary table 1.** Snapshot of the landscape of direct-to-consumer business offering secretome, EV- and, exosome-based intervention between this type of intervention between July and November 2022.

## Data Availability

The datasets used and/or analysed in this study are available from the corresponding author on reasonable request.

## Abbreviations

ATMPs: Advanced Therapy Medicinal Products
COVID: Coronavirus Disease 2019
EMA: European Medicines Agency
EU: European Union
EVs: Extracellular vesicles
FDA: U.S. Food and Drug Administration
ISEV: International Society of Extracellular Vesicles
ISEV: International Society of Extracellular Vesicles
MHRA: Medicines and Healthcare products Regulatory Agency
MSCs: Mesenchymal stem cells
