# Supplementary material for: Uncovering the gray zone: mapping the global landscape of direct-to-consumer businesses offering interventions based on secretomes, extracellular vesicles, and exosomes
Source: Stem Cell Res Ther. 2023 May 4;14:111. doi: 10.1186/s13287-023-03335-2 (PMC10156419; doi:10.1186/s13287-023-03335-2)
Supplement: Supplementary file 1 — Additional file 1. Supplementary table 1. Snapshot of the landscape of direct-to-consumer business offering secretome, EV- and, exosome-based intervention between this type of intervention between July and November 2022. [file 13287_2023_3335_MOESM1_ESM.docx]

**Supplementary table 1.** Snapshot of the landscape of direct-to-consumer business offering secretome, EV- and, exosome-based intervention between this type of intervention between July and November 2022.

| Company name | Location | Conditions treatments are advertised for | Therapy based on | URL |
| --- | --- | --- | --- | --- |
| Right Path | USA | arthritis, Parkinson`s disease`s disease, autism, diabetes, chronic diseases, Lyme disease | exosomes | [https://rightpathpainandspine.com/exosomes-therapy/](https://rightpathpainandspine.com/exosome-therapy/) |
| Life Point | USA | arthritis, Parkinson`s disease, autism, diabetes, chronic diseases | exosomes | [https://www.drtsbeck.com/blog/exosomes-therapy-versus-stem-cell-therapy-how-do-they-differ](https://www.drtsbeck.com/blog/exosome-therapy-versus-stem-cell-therapy-how-do-they-differ) |
| Infusio | USA | anti-ageing, arthritis, Parkinson`s disease, autism, diabetes, Chronic diseases, Lyme disease | exosomes | [https://www.infusio.org/treatments/exosomes-therapy/](https://www.infusio.org/treatments/exosome-therapy/) |
| ANOVA IRM | Germany | arthritis, Parkinson`s disease, autism, diabetes, chronic diseases | secretome, exosomes | <https://anova-irm.com/> |
| Perfect skin solutions | England | skin care | exosomes | [https://perfectskinsolutions.co.uk/treatments/exosomess/](https://perfectskinsolutions.co.uk/treatments/exosomes/) |
| Infusio | Germany | anti-ageing, arthritis, Parkinson`s disease, autism, diabetes, chronic diseases, Lyme disease | exosomes | [https://www.infusio.org/treatments/exosomes-therapy/](https://www.infusio.org/treatments/exosome-therapy/) |
| Honest hair restoration | USA | hair loss | exosomes | <https://www.honesthairrestoration.com/blog/all-about-exosomal-hair-restoration> |
| Progen cell | Mexico | skin care | exosomes | https://progencell.com/blog/what-you-should-know-about-exosomes-therapy-a-current-panorama/ |
| Alvi Armani | USA | hair loss | exosomes | <https://www.alviarmani.com/hair-transplant-consultation/> |
| Bay area aesthetics | USA | hair loss | exosomes | [https://www.bayareaaesthetics.net/exosomess-for-hair-loss](https://www.bayareaaesthetics.net/exosomes-for-hair-loss) |
| VIDA Medical Center | USA | skin and anti-ageing | exosomes | <https://vidaandco.net/contact-us/> |
| ISSCA | USA | skin and anti-ageing | exosomes | [https://www.issca.us/exosomes-therapy-the-secret-treatment-of-anti-ageing/](https://www.issca.us/exosome-therapy-the-secret-treatment-of-anti-aging/) |
| The John Bull Center | USA | skin and anti-ageing | exosomes | [https://www.dupageplastics.com/blog/what-are-benefits-of-exosomes-therapy/](https://www.dupageplastics.com/blog/what-are-benefits-of-exosome-therapy/) |
| Yunique Medical | USA | skin and anti-ageing | exosomes | [https://yuniquemedical.com/exosomes-therapy/](https://yuniquemedical.com/exosome-therapy/) |
| Anti aging and wellness Medical | USA | skin and anti-ageing | exosomes | [https://antiagemedical.com/exosomess/](https://antiagemedical.com/exosomes/) |
| Anti aging and wellness Medical | costa Rica | skin and anti-ageing | exosomes | [https://antiagemedical.com/exosomess/](https://antiagemedical.com/exosomes/) |
| Anti aging and wellness Medical | Mexico | skin and anti-ageing | exosomes | [https://antiagemedical.com/exosomess/](https://antiagemedical.com/exosomes/) |
| Swiss Medica | USA | Parkinson`s disease, autism | exosomes | https://www.startstemcells.com/aventure-etats-unis.html |
| Swiss Medica | Slovenia | Parkinson`s disease, autism | exosomes | <http://msci.swissmedicacrm.com/Locations/slovenia> |
| Swiss Medica | Poland | Parkinson`s disease, autism | exosomes | https://www.startstemcells.com/poland-warsaw.html |
| Swiss Medica | Austria | Parkinson`s disease, autism | exosomes | http://msci.swissmedicacrm.com/Locations/austria |
| Swiss Medica | Russia | Parkinson`s disease, autism | exosomes | <http://msci.swissmedicacrm.com/Locations/russia> |
| 300 experts | Russia | skin care | exosomes | <https://300experts.ru/znaete_li_vi/ekzosomnaya_terapiya_alternativa_ukolam_krasoti_/> |
| R3 Stem cell | Canada | arthritis, Parkinson`s disease, autism, diabetes | exosomes | <https://r3stemcell.com/canada/> |
| VitalityMD | Canada | skin and anti-ageing | exosomes | [https://vitalitymd.com/exosomess-infusion-therapy/](https://vitalitymd.com/exosomes-infusion-therapy/) |
| R3 Stem cell | USA | skin and anti-ageing | exosomes | [https://www.einnews.com/pr_news/531619473/r3-stem-cell-international-now-offering-stem-cell-and-exosomes-therapy-promotion-for-canada](https://www.einnews.com/pr_news/531619473/r3-stem-cell-international-now-offering-stem-cell-and-exosome-therapy-promotion-for-canada) |
| Ageless Radiance | Canada | skin and anti-ageing | exosomes | [https://www.agelessradiancemd.com/prp-exosomes-therapy](https://www.agelessradiancemd.com/prp-exosome-therapy) |
| Stem Cell Care | India | arthritis, Parkinson`s disease, autism, diabetes | exosomes | [https://www.stemcellcareindia.com/exosomes-therapy-in-india/](https://www.stemcellcareindia.com/exosome-therapy-in-india/) |
| CWI | India | arthritis, Parkinson`s disease, autism, diabetes | exosomes | <https://cwiindia.com/contact/> |
| Revivify | USA | hair loss | exosomes | <https://revivifymedicalspa.com/contact/> |
| Coollaser Clinic | Ukraine | skin care | exosomes | <https://coolaser.clinic/en/> |
| Aesthetix mideast | UAE | skin care | exosomes | <https://www.instagram.com/aesthetix_mideast/?hl=en> |
| Laser skin care | UAE | hair loss | exosomes | [https://www.laserskincare.ae/treatments/exosomess-therapy/](https://www.laserskincare.ae/treatments/exosomes-therapy/) |
| Hair transplant Dubai | UAE | hair loss | exosomes | [https://www.hairtransplantdubai.com/hair-loss-treatment/exosomess-for-hair-loss/](https://www.hairtransplantdubai.com/hair-loss-treatment/exosomes-for-hair-loss/) |
| Euromed clinic | UAE | skin and anti-ageing | exosomes | [https://www.euromedclinicdubai.com/dermatology/exosomes-facial-and-skin-rejuvenation/](https://www.euromedclinicdubai.com/dermatology/exosome-facial-and-skin-rejuvenation/) |
| Bedfordshire skin Clinic | England | skin care | exosomes | [https://www.bedfordskincare.com/exosomestherapy](https://www.bedfordskincare.com/exosometherapy) |
| San Diego medical centre | USA | hair loss, arthritis, Lyme disease | exosomes | [https://san-diego.exosomesscells.com/](https://san-diego.exosomescells.com/) |
| Clinic expert | Turkey | hair loss | exosomes | [https://www.clinicexpert.com/eng/exosomess-hair-before-and-after/](https://www.clinicexpert.com/eng/exosomes-hair-before-and-after/) |
| Gleam medical spa | USA | skin care, hair loss | exosomes | [https://gleammedspa.com/med-spa-services/exosomes-microneedling-facial/](https://gleammedspa.com/med-spa-services/exosome-microneedling-facial/) |
| Kellman wellness centre | USA | COVID19 recovery, anti-ageing, arthritis, Lyme disease, tendonitis, and diabetic neuropathy | exosomes | [https://www.kellmancenter.com/exosomes-therapy.html](https://www.kellmancenter.com/exosome-therapy.html) |
| For Hair | USA | hair loss | exosomes | [http://www.forhair.com/blog/exosomess-a-hair-restoration-treatment-with-awesome-potential](http://www.forhair.com/blog/exosomes-a-hair-restoration-treatment-with-awesome-potential) |
| Longevity Clinic | USA | arthritis pain | exosomes | [http://lcriusa.com/exosomes-therapy/](http://lcriusa.com/exosome-therapy/) |
| Orthocure | England | arthritis pain | exosomes | [https://orthocureclinic.com/exosomess/](https://orthocureclinic.com/exosomes/) |
| PRMedica | Mexico | arthritis, Parkinson`s disease, autism, diabetes | exosomes | <https://prmedica-inc.com/> |
| DVCSTEM | USA | arthritis, Parkinson`s disease, autism, diabetes | exosomes | <https://www.dvcstem.com/post/stem-cell-therapy> |
| The London Cosmetic Clinic | England | skin and anti-ageing | exosomes | [https://www.thelondoncosmeticclinic.co.uk/treatments/exosomes](https://www.thelondoncosmeticclinic.co.uk/treatments/exosome) |
| Mayo Clinic | USA | arthritis, Parkinson`s disease, autism, diabetes | exosomes | [https://www.mayo.edu](https://www.mayo.edu/) |
| Lucia clinic | UAE | skin and anti-ageing | exosomes | [https://luciaclinic.com/face/exoscrt-exosomess-treatment/](https://luciaclinic.com/face/exoscrt-exosomes-treatment/) |
| SAIFI | Poland | hair loss | exosomes | [https://transplantacja-wlosow.pl/en/zabiegi/exosomess-therapy-en/](https://transplantacja-wlosow.pl/en/zabiegi/exosomes-therapy-en/) |
| Dr hair tranplant | Germany | hair loss | exosomes | <https://drthair.com/de/service/behandlungen-gegen-haarausfall/> |
| Dr hair tranplant | Turkey | hair loss | exosomes | <https://drthair.com/de/service/behandlungen-gegen-haarausfall/> |
| Dr hair tranplant | Cyprus | hair loss | exosomes | <https://drthair.com/de/service/behandlungen-gegen-haarausfall/> |
| Albiraa clinic | UAE | hair loss | exosomes | [https://www.albiraaclinic.com/exosomes-therapy-in-dubai/](https://www.albiraaclinic.com/exosome-therapy-in-dubai/) |
| KRU | Germany | arthritis, Parkinson`s disease, autism, diabetes | exosomes | <https://www.sana.de/rummelsberg/medizin-pflege/kniezentrum/leistungsspektrum> |
| Dubai cosmetic Surgery | UAE | hair loss | exosomes | [https://www.dubaicosmeticsurgery.com/hair-transplant/exosomes-therapy/](https://www.dubaicosmeticsurgery.com/hair-transplant/exosome-therapy/) |
| Segova | Serbia | diabetes, neuropathy | exosomes | [https://segova.com/exosomes-therapy/](https://segova.com/exosome-therapy/) |
| R3 Stem cell | Philippine | skin and anti-ageing | exosomes | https://r3stemcell.com/philippines/locations/ |
| Premier clinic | Malaysia | arthritis, immune deficiency | exosomes | [https://premier-clinic.com/our-services/exosomes-therapy/](https://premier-clinic.com/our-services/exosome-therapy/) |
| Aliesbury clinic | Irland | hair loss | exosomes | <https://ailesburyclinic.ie/hair-restoration/> |
| MJ aesthetic | Malaysia | anti-ageing, arthritis, Parkinson`s disease, autism, diabetes | exosomes | [https://www.mj-aesthetic.com/service/regenerative-medicine-exosomess/](https://www.mj-aesthetic.com/service/regenerative-medicine-exosomes/) |
| Gem clinic | Malaysia | anti-ageing, arthritis, Parkinson`s disease, autism, diabetes | exosomes | <https://gem.clinic/treatments/long-life-booster/> |
| Ming medical | Malaysia | anti-ageing, arthritis, Alzheimer, autism, diabetes | exosomes | <https://www.mingmedical.net/magazines.php> |
| DIJOMA | Malaysia | anti-ageing | exosomes | <https://dijomaa2u.blogspot.com/p/terapiperawatan.html> |
| Xeoul clinic | Malaysia | skin and anti-ageing | exosomes | [https://xeoulclinic.com/services/exomide-stemcell-derived-exosomess/](https://xeoulclinic.com/services/exomide-stemcell-derived-exosomes/) |
| Aesthetic Scalp | USA | hair loss | exosomes | [https://aestheticscalp.com/blog/when-should-you-see-results-after-exosomess-therapy](https://aestheticscalp.com/blog/when-should-you-see-results-after-exosomes-therapy) |
| BH medical aesthetic | Singapore | skin care, hair loss | exosomes | <https://www.bhmedicalaesthetics.com/contact> |
| Cheongdam skin | Singapore | skin care | exosomes | [https://cheongdamskin.com.sg/product/exosomes-magic-skin/](https://cheongdamskin.com.sg/product/exosome-magic-skin/) |
| Veritas | Singapore | skin care | exosomes | [https://veritas.com.sg/exosomes-stem-cell-therapy-for-hair-growth/](https://veritas.com.sg/exosome-stem-cell-therapy-for-hair-growth/) |
| Regeneration center | Thailand | anti-ageing, arthritis, Parkinson`s disease, autism, diabetes | exosomes | [https://stemcellthailand.org/exosomess-extracellular-vesicles/](https://stemcellthailand.org/exosomes-extracellular-vesicles/) |
| Hair tran | Thailand | hair loss | exosomes | <https://www.hairtranclinic.com/en/non-surgical-vs-surgergical/> |
| KRU Wellness | Thailand | anti-ageing, arthritis, Parkinson`s disease, autism, diabetes | exosomes | <https://kruwellness.com/> |
| Svenson | Malaysia | hair loss | exosomes | <https://www.svensonhair.com.my/promotions/ap-hair-loss-therapy> |
| The esthetic clinic | India | hair loss | exosomes | <https://www.theestheticclinic.com/cosmetic/hair-loss-treatment/stem-cell-hair-transplant.html> |
| Boutique clinic | USA | skin care, hair loss | exosomes | [https://boutiqclinic.com/exosomess/](https://boutiqclinic.com/exosomes/) |
| Concierge MD | USA | Covid recovery | exosomes | [https://conciergemdla.com/blog/conditions-exosomes-therapy-can-treat/](https://conciergemdla.com/blog/conditions-exosome-therapy-can-treat/) |
| Jivaka | south Korea | skin care, hair loss, anti-ageing | exosomes | [https://beauty.jivaka.care/products/exosomes-skincare-chang-dermatology-clinic](https://beauty.jivaka.care/products/exosome-skincare-chang-dermatology-clinic) |
| Optimal health wellness | USA | anti-ageing, arthritis, Parkinson`s disease, autism, diabetes, Chronic diseases, Lyme disease | exosomes | [https://opthealthwellness.com/how-long-does-it-take-exosomes-treatment-to-work/](https://opthealthwellness.com/how-long-does-it-take-exosome-treatment-to-work/) |
| Genesys laser clinic | USA | skin care | exosomes | [https://genesyslaserclinic.com/exosomes/](https://genesyslaserclinic.com/exosome/) |
| EVO | Germany | anti-ageing, chronic diseases disease | exosomes | [https://evofrankfurt.com/portfolio-item/exosomess](https://evofrankfurt.com/portfolio-item/exosomes) |
| Quito | Ecuador | skin care and anti-ageing | exosomes | <http://www.medicentergt.com/> |
| Organicell | Guatemala | skin care and anti-ageing | exosomes | <https://organicell.com/> |
| Organicell | USA | skin care and anti-ageing | EVs | https://organicell.com/ |
| Apollo hospital | India | cancer, heart disease, skin care | exosomes | <https://apollohospitals.com/> |
| Ageless 3.0 | USA | skin and anti-ageing | exosomes | [https://ageless30.com/prp-exosomes-therapy](https://ageless30.com/prp-exosome-therapy) |
| Body RX | USA | skin and anti-ageing | exosomes | [https://www.bodyrxantiaging.com/sports-medicine/stem-cell/body-rx-anti-ageing-announces-stem-cell-treatments-now-available-miami-locations/](https://www.bodyrxantiaging.com/sports-medicine/stem-cell/body-rx-anti-aging-announces-stem-cell-treatments-now-available-miami-locations/) |
| Drop hydration | USA | arthritis | exosomes | <https://driphydration.com/stem-cell-iv-therapy/> |
| Prmedica | Mexico | skin care | exosomes | <https://prmedica-inc.com/> |
| Giostar | Mexico | skin and anti-ageing, arthritis, Parkinson`s disease, autism, diabetes | exosomes | [https://giostarmexico.com/news/Exosomess-Treatment](https://giostarmexico.com/news/Exosomes-Treatment) |
| Holisticbiospa | Mexico | skin and anti-ageing, arthritis, Parkinson`s disease, autism, diabetes | exosomes | <https://www.holisticbiospa.com/stem-cell-therapy/> |
| R3 international, Cancun | Mexico | anti-ageing, arthritis, Parkinson`s disease, autism, diabetes, Chronic diseases, Lyme disease | exosomes | [https://markets.businessinsider.com/news/stocks/r3-international-now-including-exosomess-with-stem-cell-therapy-program-for-autism-in-mexico-1029422555](https://markets.businessinsider.com/news/stocks/r3-international-now-including-exosomes-with-stem-cell-therapy-program-for-autism-in-mexico-1029422555) |
| R3 international, Tijuana | Mexico | anti-ageing, arthritis, Parkinson`s disease ,autism, diabetes, Chronic diseases, Lyme disease | exosomes | https://markets.businessinsider.com/news/stocks/r3-international-now-including-exosomess-with-stem-cell-therapy-program-for-autism-in-mexico-1029422555 |
| R3 international, Mexicali | Mexico | anti-ageing, arthritis, Parkinson`s disease, autism, diabetes, Chronic diseases, Lyme disease | exosomes | https://markets.businessinsider.com/news/stocks/r3-international-now-including-exosomess-with-stem-cell-therapy-program-for-autism-in-mexico-1029422555 |
| Cosmetic dental | south Africa | skin care | exosomes | [https://capetowndentist.co.za/exosomess/](https://capetowndentist.co.za/exosomes/) |
| MJ Dermland | south Korea | skin care and anti-ageing | exosomes | <https://koreandramaland.com/listings/mj-dermatology-clinic/> |
| De felipe Barcelona | Spain | skin and hair loss | exosomes | <https://www.defelipe.com/medicina-regenerativa-barcelona/exosomas-barcelona/> |
| De felipe Madrid | Spain | skin and hair loss | exosomes | https://madrid.defelipe.com/medicina-regenerativa-madrid/exosomas-madrid/ |
| Exolife | Spain | skin and hair loss | exosomes | <https://www.clinicaexolife.com/> |
| Beauty med | Spain | skin and hair loss | exosomes | [https://www.beautymed.es/cellgenic-exosomess-revoluciona-la-medicina-regenerativa-24573.php](https://www.beautymed.es/cellgenic-exosomes-revoluciona-la-medicina-regenerativa-24573.php) |
| Barber surgeons | USA | hair loss | exosomes | [https://barbersurgeonsguild.com/hair-restoration/exosomes-therapy/](https://barbersurgeonsguild.com/hair-restoration/exosome-therapy/) |
| DR. Yasamin Zandi | Iran | hair loss | exosomes | <https://dryasamanzandi.com/clinic/> |
| Europe clinic | UAE | hair loss | exosomes | <https://www.instagram.com/drweberclinic/> |
| Helia clinic | Iran | hair loss | exosomes | <https://heliaclinic.com/stem-cell-hair-transplantation/> |
| Stem cell revolution | Iran | arthritis | exosomes | https://stemcellrevolution.com/treatment-centers/iran/ |
| Cosmetic laser solution | USA | skin care | exosomes | [https://www.cosmeticlasersolutions.net/what-is-the-exosomes-facial/](https://www.cosmeticlasersolutions.net/what-is-the-exosome-facial/) |
| Aestha clinic | India | skin and anti-ageing, arthritis | exosomes | <https://www.facebook.com/aesthaclinic> |
| FACE Med Store | USA | hair loss | exosomes | [https://facemedstore.com/blogs/blog/is-exosomes-therapy-effective-for-hair-loss-reviews](https://facemedstore.com/blogs/blog/is-exosome-therapy-effective-for-hair-loss-reviews) |
| SMILe EST | Turkey | skin and anti-ageing | exosomes | [https://www.smileest.com.tr/en/exosomes-stem-cell-therapy/](https://www.smileest.com.tr/en/exosome-stem-cell-therapy/) |
| Este Grande | Turkey | hair loss | exosomes | [https://www.este-grande.com/en/exosomess-a-hair-restoration-treatment-with-awesome-potential/](https://www.este-grande.com/en/exosomes-a-hair-restoration-treatment-with-awesome-potential/) |
| NAD Treatment Centre | USA | skin and anti-ageing, neuropathy | exosomes | [https://www.nadtreatmentcenter.com/exosomes-therapy/](https://www.nadtreatmentcenter.com/exosome-therapy/) |
| Iscelli | USA | skin and anti-ageing, hair loss | exosomes | [http://iscelli.com/exosomess/](http://iscelli.com/exosomes/) |
| Patt md | England | hair loss | exosomes | [https://www.pattmd.com/dr-patts-blog/stem-cell-hair-transplant-regrowth-and-the-role-of-exosomess](https://www.pattmd.com/dr-patts-blog/stem-cell-hair-transplant-regrowth-and-the-role-of-exosomes) |
| Tijuana and Cancun | Mexico | skin and anti-ageing, arthritis, Parkinson`s disease, autism, diabetes | exosomes | [https://stemcelltreatmentclinic.com/exosomes-therapy-in-mexico-tijuana-and-cancun/](https://stemcelltreatmentclinic.com/exosome-therapy-in-mexico-tijuana-and-cancun/) |
